# Opening the black box of article retractions: exploring the causes and consequences of data management errors

**DOI:** 10.1098/rsos.240844

**Published:** 2024-12-18

**Authors:** Marton Kovacs, Marton A. Varga, Dominik Dianovics, Russell A. Poldrack, Balazs Aczel

**Affiliations:** ^1^ Doctoral School of Psychology, ELTE Eotvos Lorand University, Budapest, Hungary; ^2^ Institute of Psychology, ELTE, Eotvos Lorand University, Budapest, Hungary; ^3^ Department of Psychology, Stanford University, Stanford, CA, USA

**Keywords:** retraction, honest error, research data management

## Abstract

The retraction of an article is probably the most severe outcome of a scientific project. While great emphasis has been placed on articles retracted due to scientific misconduct, studies show many retractions are due to honest errors. Unfortunately, in most cases, retraction notices do not provide sufficient information to determine the specific types and causes of these errors. In our study, we explored the research data management (RDM) errors that led to retractions from the authors’ perspectives. We collected responses from 97 researchers from a broad range of disciplines using a survey design. Our exploratory results suggest that just about any type of RDM error can lead to the retraction of a paper, and these errors can occur at any stage of the data management workflow. The most frequently occurring cause of an error was inattention. The retraction was an extremely stressful experience for the majority of our sample, and most surveyed researchers introduced changes to their data management workflow as a result. Based on our findings, we propose that researchers revise their data management workflows as a whole instead of focusing on certain aspects of the process, with particular emphasis on tasks vulnerable to human fallibility.

## 1. Introduction

Whenever there is a plane crash, aviation experts rush to explore the reasons behind the catastrophe. Considerable efforts are made to discover and disclose every seemingly minute detail that could have played a role in the accident. The ultimate goal of this investigation is not to find someone to blame for the event but to acquire knowledge to create better error-prevention systems that decrease the chance of another crash in the future. Article retractions in the world of scientists are the crashes of research projects. Although retractions help clear the scientific literature of inaccurate reports, they should not be ignored as they contain valuable information regarding the sources of errors. Nevertheless, unlike in the field of aviation, the research community rarely uses these cautionary examples to update their research workflows to avoid similar mishaps and their consequences. The present study takes a step towards systematically exploring research data management (RDM) errors that contribute to retractions from the authors’ viewpoint. A research programme in this area should enhance researchers’ awareness of these errors and help them prevent and address errors before submitting their manuscripts.

RDM is a complex, multifaceted process that aims to ensure research data integrity and long-term usability. It encompasses all stages of the research life cycle related to data, from planning the data collection to reporting the results. Good RDM practices are fundamental for establishing the cumulative nature of science by allowing other researchers to verify findings and build upon them [1]. By ensuring that research data are findable, accessible, interoperable, and reusable (aligned with the FAIR principles [2]), RDM benefits various stakeholders within the scientific ecosystem by efficiently using valuable research outputs.

During RDM, basic tasks such as merging datasets, transforming variables, or excluding specific data points are prone to errors if conducted manually and without proper safeguards, irrespective of prior research experience. A recent survey among psychology researchers indicated that for most laboratories, RDM errors happen with a low frequency [3]. This finding probably underestimates the actual frequency of RDM errors within psychology, given that respondents could only disclose errors they detected. While RDM errors were found to be infrequent, nearly a quarter of the respondents admitted that the most severe errors led to major consequences such as significant time and money loss, damage to professional reputation, or influenced even the conclusions of the study.

Errors that are not detected before submission can result in the retraction of a paper, which is one of the most serious negative outcomes of a research project. Several researchers who openly admitted their errors and decided to ask for the retraction of their work vividly described the distressing realization of the error, the fear of losing professional credibility and funding and the embarrassment in front of their coauthors [4,5]. Only a few researchers choose to be as open as this about their experience with the retraction due to honest errors, leaving the silent majority whose experiences we know little about. Different estimates exist on what proportion of the retractions are due to honest errors mainly based on the content of retraction notices, a short summary of the reasons behind the retraction published at the time of retraction. The exploration of retracted papers in the Medline bibliographic database between 1982 and 2002 classified 61.8% of the reasons behind retractions as errors [6]. A later study that was conducted using the same database used a more detailed classification method and showed that 40% of the retractions were due to honest errors on the part of the authors (such as analysing the wrong cell line or using incorrect data) or non-replicable findings [7]. Another study that examined multiple published resources (e.g. results of the investigation conducted by institutional research integrity offices) on retractions besides the retraction notices to further specify their classification method estimated that 20% of the cases are due to honest errors [8]. The significant variations in the estimates could stem from the diverse datasets used by the authors for calculation, the unclear explanations in the retraction notices about the reasons for retractions, or the methods employed to categorize the causes of retractions [9].

When such a crushing event occurs, it is common to look for someone to blame and attribute the error to the personal failures of the perpetrator [10]. However, since researchers are human, fallibility is inherent, as demonstrated by the fact that human errors occur in even the most high-stakes situations, such as human space flight [11] or surgery [12]. Our cognitive capabilities are finite; fatigue can set in, and concentration can fade. Moreover, the possibility of making an error is further increased by the pressure to publish. When researchers from the fields of psychology and neuroscience were asked to name the limitations of their RDM practices, they reported the lack of time as one of the most prominent limitations [13,14]. To efficiently decrease the occurrence of procedural errors and mitigate their adverse outcomes, errors should be seen as flaws of the RDM system that was designed to prevent them [10,15]. However, to construct such systems, we must understand when and how RDM errors occur.

Retraction notices have the potential to provide information about the circumstances that led to the retraction in order to construct effective prevention methods. However, several empirical investigations of published retraction notices indicated that these documents currently fall behind this promise [16–18]. For example, a study exploring a random selection of more than 2000 retraction notices found that around 10% of the notices did not contain sufficient information, even about the broad reasons behind the retraction [18]. Retraction notices are often the result of an extended discussion between the authors, their affiliated institution and the journal. Quite often, not all parties involved agree on the decision to retract or have differing interests in these disputes. Thus, several factors can influence the credibility and informativeness of the retraction notice regarding what exactly happened, who called for the retraction and who takes responsibility for it.

In summary, the available information about most retractions is not informative enough to understand how things went wrong in the project. Therefore, in this explorative study, we contacted the authors of papers retracted due to procedural RDM errors to better understand their perspective on the error and the retraction process in general. We explored the type and cause of the error, the stress the authors experienced due to the retraction and the changes they made to their RDM workflow as a result.

## 2. Methods

### 2.1. Participants

Our sample was based on a list of retracted papers (*n* = 36 773) extracted from the Retraction Watch Database [19]. We only included papers retracted due to RDM errors, as indicated by tags assigned by the Retraction Watch team, for each paper based on its retraction notice. Also, we excluded any duplicates and papers that did not have a corresponding reference ID (such as DOI or PubMedID) in the Retraction Watch database. At the end of this prescreening, we had 5816 retracted papers left in our collection. The data collection was approved by the Ethics Board of ELTE, Hungary, with the reference number 2022/301 (for more details, see https://osf.io/nymza/).

Next, we used the PubMed API and a custom crawler script to extract the email addresses of the corresponding authors from the retracted papers. In cases where multiple email addresses were returned from a paper, we retained all of them for our survey. Eventually, we contacted 6680 email addresses extracted from 5041 retracted papers. Further details about the sample creation method can be found in the electronic supplementary material.

### 2.2. Materials

In our survey, first, we asked researchers whether they agreed that the retraction of their paper was caused by RDM errors (answer options: ‘not at all’, ‘partially’, ‘it was the main reason’). The survey was terminated for respondents who thought that their paper was not at all retracted due to RDM errors. Next, we asked respondents about the nature of the RDM error that was the most responsible for the retraction. We used the RDM error taxonomy developed by Kovacs *et al*. [3] that describes 20 data management error types (such as *data coding error*, *ambiguous naming/defining of data*, *incorrect software or hardware settings*) and 15 possible causes (such as *bad or lack of planning*, *miscommunication*, *time pressure*) for the errors. The respondents could only choose one error type and the corresponding cause behind it. If none of the response options applied to their case, respondents had the option to provide free-text responses.

We also asked the respondents to indicate how much stress they experienced due to the retraction of their paper on a seven-point Likert-type scale (0—no stress at all, 6—extreme stress). Next, we asked the respondents whether they changed anything in their research workflow because of the retraction (Yes or No). The respondents also had the option to share any additional information regarding their experience with the retraction in free text. Finally, the respondents could provide any comments as free text at the end of our survey.

### 2.3. Procedure

We used the Qualtrics email sender service to contact the authors in our sample. The recruitment letter with a link to the survey was sent out in five batches between 9 May 2023 and 6 July 2023. Approximately 1 week later, a reminder was sent to the participants who had not responded to our survey and had not opted out. The recruitment letter and further information about the data collection procedure can be found in the electronic supplementary material.

### 2.4. Data preprocessing

For the questions regarding the error types and their causes, some respondents found none of the provided categories applicable. Thus, 16 respondents described their errors in free-text responses and 7 of them reported the causes of the errors as free-text responses. To calculate the frequencies of each category, we first grouped these responses based on their central features. During the grouping process, some responses were excluded as they were irrelevant to the given question or contained insufficient information. In the end, we could assign all the remaining free-text responses (5 for the error types and 4 for the causes) to one of the already existing groups. A more detailed description of the data preprocessing can be found in the electronic supplementary material.

### 2.5. Qualitative analysis

We used thematic analysis [20] to identify recurring themes in the free-text responses to the question regarding the authors’ experiences with the retraction process and any further comments. During this analysis, we were mainly interested in the content of the identified themes and not their prevalence. Upon the first reading of the responses, we realized that the authors discussed many different aspects of the RDM error and the retraction process. Thus, to ensure coherence and consistency in the identified themes, we deviated from our preregistered plan of analysing all responses to these questions without a predefined scope. Instead, we limited the focus of the thematic analysis by first identifying multiple research questions based on previous literature and the content of the responses. We formulated the following two questions of interest: (i) *What practical changes did the authors introduce to their research workflow as a result of the error and/or the retraction?* (ii) *What practical changes do the authors recommend to journals regarding the retraction process?* The thematic analysis was mainly carried out by one of the authors (M.K.). In cases where the grouping of the free-text responses was not clearly classifiable, M.K. consulted B.A. During the thematic analysis, first, we flagged each free-text response that contained information regarding one of our two research questions. Second, the relevant part of the response to the question was extracted. Third, we assigned a short-text code to the extract that summarized the content of the extract from the viewpoint of the given question. Fourth, we assigned each code to a higher-level theme in an iterative manner. More information about the qualitative analysis can be found in the electronic supplementary material.

## 3. Results

### 3.1. Number of responses

**Figure 1.**
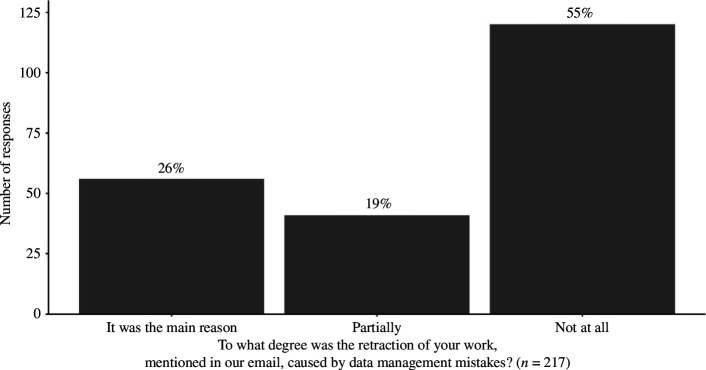
Number and proportion of responses to the question asking whether data management errors caused the retraction.

Out of the 6682 emails sent in 5 batches, 1367 bounced, 71 were duplicates and 5244 were delivered. Out of these, 246 (response rate: 4.69%) respondents started to fill out our survey. From the analysis, we excluded 5 respondents who did not consent to our informed consent form, 23 respondents who did not respond to the mandatory questions, 1 respondent who indicated that they were not the author of the paper we reference in the recruitment letter and 120 respondents who indicated that RDM errors did not cause the retraction of their paper (figure 1). By the end of the exclusions, we had responses from 97 remaining respondents for the main analysis. Some responses to free-text questions were excluded during the data preprocessing because they lacked enough relevant information to answer the question. As a result, we separately note the number of all responses for each result. The median response time was 7.6 min.

### 3.2. Types of errors and their causes

**Figure 2.**
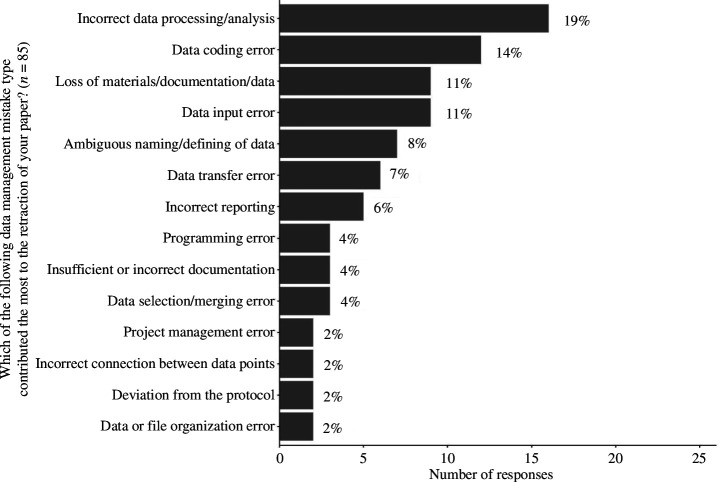
The frequency and percentages of the RDM error types that led to the retraction of the paper. After excluding the missing and irrelevant responses, 85 responses remained in our sample.

We found 18 different error types that led to the retraction of a paper in our sample. The four most frequently occurring error types were *Incorrect data processing/analysis* (*n* = 16), *Data coding error* (*n* = 12), *Data input error* (*n* = 9), and *Loss of materials/documentation/data* (*n* = 9). Figure 2 shows the percentages and counts of all the error types in our sample.

**Figure 3.**
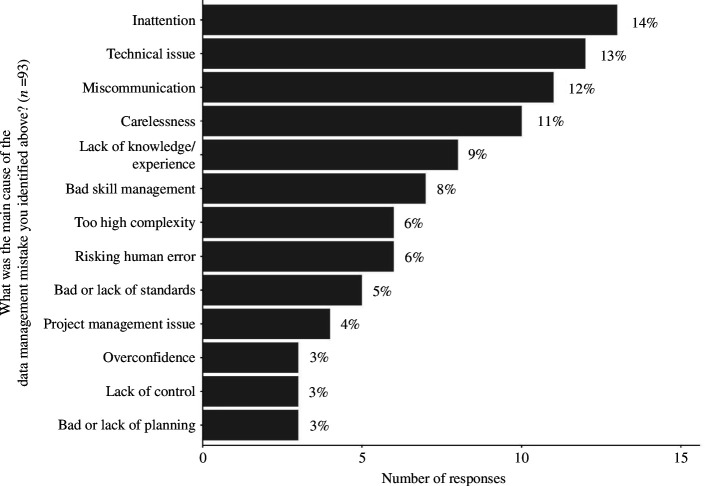
The frequency and percentages of causes that led to the retraction of the paper. After excluding the missing and irrelevant responses, 93 responses remained in our sample.

The respondents identified 15 different potential causes behind these errors. The most frequent causes were: *Inattention* (*n* = 13), *Technical issue* (*n* = 12), and *Miscommunication* (*n* = 11). Figure 3 shows the counts and percentages of the suspected causes behind the data management errors.

**Figure 4.**
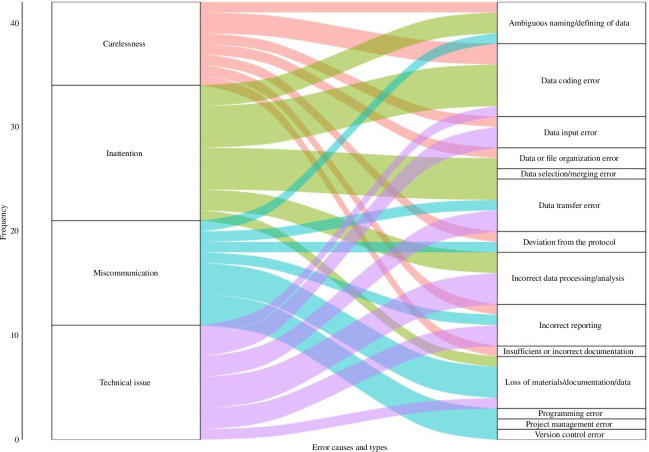
Relationship between the most frequent causes and the RDM error types. For this figure, we used 42 responses as we only included errors caused by the four most frequent causes of errors.

To identify the error types most frequently caused by various factors, we plotted the causes occurring in more than 10% of the cases along with their associated error types, as shown in figure 4. In the figure, the thickness of the lines represents the frequency with which a cause leads to a specific error type, while the thickness of each box represents the frequency with which each cause or error type occurs.

### 3.3. Experiences with the retraction

**Figure 5.**
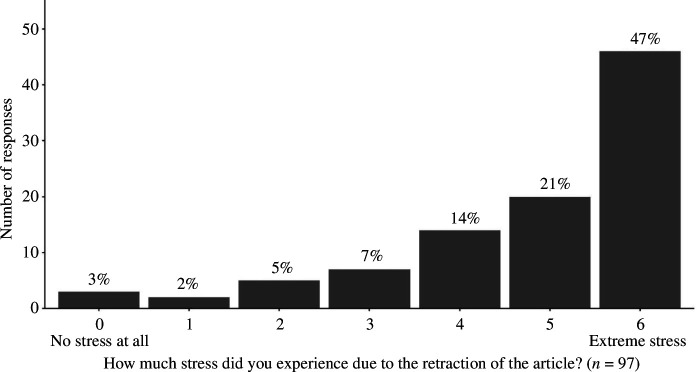
The frequency and percentages of the stress that the retraction caused to the authors on a seven-point Likert-type scale. The *X*-axis shows the scale, while the *Y*-axis shows the number of responses.

Most of the respondents (47%, 46 out of 97) indicated on a seven-point Likert-type scale ranging from 0 to 6 that the retraction of their article caused them extreme stress (median = 5, IQR = [4, 6]). Figure 5 shows the percentages and counts of each response option. In accordance, most of the respondents (76%, 74 out of 97) changed their research workflow because of the retraction, and only 23% (24 out of 97) kept their practices the same.

### 3.4. Lessons learned regarding research data management workflow

Based on the free-text responses, we summarized all the different practices that respondents introduced to their RDM workflow as a result of their experience with the retraction. We identified 14 recurring topics, which are listed in table 1.

**Table 1.** List of research data management practices that researchers introduced to their workflow as a result of the retraction.

practice	explanation
being selective of co-authors	holding the person responsible for the error accountable or being more selective of who to collaborate with in the future
being selective of materials	vetting data for secondary data use more properly and not trusting the published literature blindly
consulting experts	consulting with statistical experts regarding the analysis
creating adequate data storage and backups	making sure that the data have centralized backups, stored for long term, stored in a well-structured manner, or remotely shared before publication
not admitting to errors	never cast doubt on the conclusions or admit to errors publicly
formatting and structuring data and/or files	using database systems and making sure that the content of the datafiles allows for easily spotting errors in data input
being aware of one’s own limitations	not collaborating on projects that one is not an expert in
paying attention	concentrating on data management tasks that are prone to human error and taking the time with them
providing documentation	providing detailed documentation of the data management steps and code
providing training	providing adequate training, supervision and more hands-on management to team members
replicating	replicating the study results before submission
share data	sending the raw data to journals for checking
use stricter protocols	using stricter data management protocols
validate	double-check the raw data and the code, ask independent reviewers/team members/experts to check them, compare results to the results of similar and published studies and introduce multiple validity checks

We also summarized the respondents’ suggestions to journals regarding the retraction process based on their own experiences. The three main suggestions were the following: (i) journals should take a greater role in validating the data and analysis; (ii) retraction notices should be more informative regarding the error; and (iii) journals should have clearer retraction policies to avoid confusion about whether certain errors lead to retraction or just a corrigendum.

## 4. Discussion

Proper data management is integral to producing rigorous and replicable scientific findings. Failure to do so can lead to errors and, eventually, to the retraction of a paper if they are not caught before publication. Therefore, learning more about what these errors are and what might cause them is essential for developing effective prevention methods.

This study aimed to explore the circumstances behind honest errors that led to the retraction of a paper. Our goal was to uncover what happened and why from the authors’ perspective. The study provides three main findings. First, the authors’ personal accounts testified that the causes and consequences of article retractions are not extractable solely from retraction notices or other publicly available sources. The authors’ personal experience plays a crucial role in understanding the complexity of retractions. Second, instead of pointing to a few error types, the survey results indicated that just about any type of data management error can lead to retraction. Third, inattention was the most frequent cause behind these errors, highlighting the limitations of human capacity as a main challenge to tackle. In the following sections, we will discuss these findings in more detail and then explore potential ways for both researchers and journals to prevent, detect or mitigate the effects of honest errors. Finally, we will outline the need for an open laboratory and a scientific culture that values error correction and encourages those who are not afraid of admitting their failures.

### 4.1. Looking beyond retraction notices

Previous studies focusing on retractions have primarily utilized large databases of retracted papers to explore their prevalence and the general reasons for retraction as stated in the retraction notices [21]. While the statistics of retractions can provide descriptive information about the current state of scholarly publications, they offer little in the form of specific causes of retractions and their effects on the authors. To gain these valuable insights, readers must look for self-published retraction stories and interviews with authors whose papers have been retracted. However, compared to the number of retractions due to honest errors, the number of such reports is infinitesimal.

The qualitative analysis of free-text responses in our survey revealed that the circumstances surrounding the errors—specifically what happened, why they erred, who discovered the error, how this experience affected the researchers involved and what lessons they learned from it—are far more complex than what a retraction notice would reveal. Our survey uncovered personal hurdles and situations—such as moving to different institutions, key personnel in data management taking long medical leaves and souring relationships between co-authors—that we all experience in our work lives but rarely consider in terms of their effects on the credibility of our findings. Moreover, an important reason for exploring ways to prevent retractions could be the emotional toll it takes on the researchers involved, as 47% of our respondents indicated that they experienced extreme stress due to the retraction of their article. Such insights cannot be extracted from the retraction notices alone, highlighting the need for looking at the first-hand experiences of the researchers involved.

### 4.2. Data management error types

Our respondents reported a wide range of RDM errors that eventually caused the retraction. They disclosed 18 different error types out of 20 identified by a previous study with a larger sample of researchers from the field of psychology [3]. This suggests that most RDM error types have the potential to lead to serious consequences. Importantly, these error types cover all stages of the RDM process, indicating that it is not enough to introduce a few good data management practices that target certain aspects of the data management pipeline but the whole data management process needs to be reviewed and carefully controlled to avoid the occurrence of such honest errors. Among the many types of errors, the most frequently reported one was *Incorrect data processing/analysis* which encompasses errors such as calculating the wrong statistic, incorrect application of formulas and other statistical errors. However, many other retractions were due to the incorrect coding or recoding of the variables, data loss or data entry errors. Thus, seemingly minor inaccuracies in the data management workflow can lead to erroneous conclusions. For example, one of our respondents noted in a free-text response that their error was caused by the format in which they store large numbers that contain many zeros. If there are no digit grouping separators, it is hard to spot one fewer or more zero when entering the data manually.

### 4.3. Causes for committing an error

While our results indicate that various factors can lead to data management errors, we would highlight the most frequently reported cause: inattention. Our cognitive capacity is a limited resource that we cannot simply enhance. To avoid errors due to inattention, we must carefully examine our entire RDM workflow and implement safety measures that prevent errors even if the analyst is inattentive. This reasoning aligns with the human error framework [22], which accepts individual actors’ shortcomings as a given and focuses on building systems that prevent errors. As mentioned in the introduction, aviation companies rely on similar system-centred approaches to ensure passenger safety. We would be rightfully outraged if the safety of all passengers on a commercial aeroplane depended on how much coffee the pilot had consumed before the flight or whether they had personal problems. In RDM, we risk human fallibility where tasks are performed manually, processes are not standardized and validation procedures require improvement.

### 4.4. Possible solutions to prevent, detect and mitigate errors

By exploring these errors, our ultimate goal is to help other researchers avoid making them in the future. However, being aware of the possibility of such errors is only the first step. Changing someone’s data management habits may be the real challenge. This often requires learning new skills and techniques, carefully examining the current data management workflow and convincing our collaborators to act similarly. Given that the retraction process was a highly stressful experience for the authors in our sample, it is possible that our respondents were motivated to change their workflow for the better. Three-quarters of the researchers in our sample reportedly updated their regular workflow as a result of the retraction.

Learning the necessary RDM skills takes time and effort on the part of researchers. Fortunately, many academic institutions offer RDM services, such as consultations or training provided by data librarians or research software engineers. However, a broader institutional commitment and increased global community-building efforts are needed to fully harness the potential of these services, as indicated by a recent survey of academic library workers [23]. Incorporating the diverse perspectives of various stakeholders in research data requires greater efforts to establish both field-specific and cross-disciplinary best practices and minimum requirements. These steps are essential to prevent valuable research outputs from becoming research waste.

Most of the error prevention and detection methods implemented by the surveyed researchers (e.g. validity checks, structured and well-documented data and code files; see table 1 for the full list) are in line with the recommendations of the published literature (e.g. [24,25]). As our results suggest, most error types identified by a previous study among psychologists can lead to retraction [3]. Consequently, researchers should focus on developing a well-thought-through data management pipeline instead of incorporating some good data management practices into their workflow. Borghi *et al*. [26] suggested using an RDM maturity scale for researchers to determine whether their RDM practices are robust enough at each stage of the data management process. RDM practices implemented in an ad hoc manner have a greater potential to lead to errors. In contrast, if the research teams have a well-designed RDM system in place, errors are less likely to occur in general due to human fallibility. In table 2, we list some concrete suggestions for good data management practices that researchers should consider incorporating into their workflow if they wish to reduce the likelihood of committing serious RDM errors.

**Table 2.** A non-exhaustive list of good data management practices for preventing, detecting and mitigating data management errors.

strategy	data management practice	potential benefits
structuring	well-formatted data files and directory structures; informative and standard file and variable naming	ensures that the correct datafiles and variables are analysed; prevents data loss; facilitates data integrity checks
documentation	providing project README, metadata files and data codebooks; commenting analysis code	prevents information loss; decreases the chance of miscommunication; enhances reproducibility by others; facilitates validation
versioning	using version control (e.g. Git)	easier to track causes of errors; prevents version conflicts and information loss
validation	writing unit tests; inviting independent reviewers; consulting with experts	decreasing the chance of human error; ensuring the replicability of the findings
archiving	storing data, code and documentation in third-party repositories	provides proof of honesty in case of a retraction; facilitates replication of the results
culture	having a non-punitive approach to dealing with errors; discussing errors with team; having laboratory policies for error handling; standardized RDM workflow for the laboratory; creating an environment which facilitates quality RDM	increases the chance that errors are reported; facilitates validation

While researchers can decrease the prevalence of RDM errors and detect them before publication, one could argue that journals have a definite responsibility to ensure the credibility of scientific findings through the publication process. Indeed, multiple respondents mentioned that they felt the error should have been caught during peer review. However, there are several reasons why it is highly unlikely that data management errors are caught at this stage. First, most reviewers work free of charge, and due to the rising number of publications, they are already quite overwhelmed. Thus, they cannot be expected to take the considerable amount of time it takes to carefully examine every step of the data preprocessing and analysis. Second, while a growing number of journals require authors to transparently share the data and the analysis code, for most journals, these requirements are still not part of their submission guidelines, rendering it impossible to properly check for errors. Moreover, recent studies assessing the quality of the openly shared data found that these data were often not compliant with the FAIR data principles [2], making it harder for the researchers to replicate the data management and analysis procedures [27,28].

Fortunately, several proposed solutions in the published literature could mitigate both of these obstacles. One possible method is for authors to create a reproducible analysis workflow that incorporates the data preprocessing, the analysis, and the reporting of the results in a single capsule that can replicate the findings with the click of a button [29]. These methods considerably shorten the time needed to reproduce the results by eliminating the need to collect the necessary files for reproducing the results, understand their structure and relation and create an environment with the appropriate packages installed. There are many tutorial papers available on how to create such analytical pipelines in general and specifically in certain scientific fields. However, errors can still occur in reproducible workflows. To further reduce the likelihood of such issues, journals could employ statistical reviewers with the sole purpose of ensuring the reproducibility of the published findings. While statistical reviews are found to be extremely useful for identifying statistical errors in submitted manuscripts [30], the number of journals that regularly employ statistical reviewers is estimated to be low among biomedical journals [31] and even lower in psychology journals [32]. Some of our respondents reported that they invited statistics experts to review their analysis before submission as a consequence of their retraction. Finally, automating data management tasks such as data coding or providing sufficient documentation can help decrease the chance of human error. Although large language models are currently not tailored for RDM, they could help researchers in data management and analysis code validation and standardization tasks with human oversight [33].

### 4.5. Encouraging honesty and the importance of sharing errors

Even after employing several methods to prevent them, errors may still occur. How we handle these situations as individuals and as the research community can make a difference in how likely it is that others own up to their errors instead of hiding them. While some respondents in our sample were encouraged by their collaborators or colleagues to admit the error and contact the journal, others noted that their peers called them self-saboteurs for retracting their own work: ‘science will self-correct’. Influenced by their experience with the error and the retraction procedure, some authors were positively affirmed in their position to admit to errors. One respondent expressed: ‘It was extremely sad, when we found out about the mistake and had to retract. However, I was very positively surprised how the journal was understanding our situation and gave us a chance to resubmit, when we had corrected for the mistake’. It is important to note that the journal’s attitude towards the authors’ retraction request significantly influenced how they perceived the overall experience. Other respondents had much more unpleasant experiences and, sadly, swore never to call for a retraction again. One respondent stated they instruct their students to never admit errors or question conclusions. This is quite unfortunate since the culture of a research laboratory can have a great impact on how its members approach science. Many researchers call for implementing an open laboratory culture about errors [15,34]. Since the retraction of her paper, Julia Strand has had laboratory meetings periodically where the members discuss the errors they have made recently, and they record the lessons they learned from them [10].

The fear of blame and the anticipated shame from the research community is an important deterrent to doing the right thing and calling for self-retraction when an error is discovered. Dorothy Bishop vividly describes the nervousness of seminar attendees when she asked them whether a hypothetical researcher should come clean after realizing an error that invalidated their published findings [35]. However, as she rightly points out, empirical observations show that, in real life, researchers who decide to step out often do not experience repercussions, and contrary to their expectations, they can even experience a slight increase in the number of their citations [36]. To change researchers’ perceptions of the potential drawbacks of admitting their errors, clear policies on reporting retractions are needed. For example, many authors call for the standardization of retraction notices, emphasizing the importance of reporting who called for the retraction [37]. Fanelli [38] suggests that journals should allow authors to sign their self-retraction to clearly signal the retraction was due to an honest error and not research misconduct. Another possible point of contention between authors and journals lies in the inconsistency of journals’ policies regarding when to call for a retraction. An investigation of the retraction policies of 15 major science, technology and medicine publishers and four dominant publishing-related organizations found that the definitions of retractions, errata, corrigenda and expressions of concerns remarkably deviated from the Committee on Publication Ethics guideline [39], which is considered the standard for scientific publishing [40]. Whether a retraction, partial retraction or corrigendum should be issued in the case of unreliable findings due to honest error was often found to be inadequately documented and left unclear. Not surprisingly, some researchers interviewed by Hosseini *et al*. [36] reported that they expected the journal to issue a correction when they contacted them regarding the error. Similarly, some of our respondents reported being surprised to see their paper being retracted. For other papers with similar errors, journals only issued corrections, as the errors only affected a small portion of the results.

While it is important to revise current retraction policies to encourage authors to step forward in cases where they discover an error that invalidates the findings, there is a need to place a greater emphasis on error prevention in contrast to post-publication correction. By preventing the errors from happening or detecting them before publication we can avoid (i) a considerable amount of frustration that comes with the retraction process on the part of the authors, their affiliated institutions and the journals alike; (ii) a waste of resources by all parties involved as the retraction process is often a lengthy procedure consisting of an exhaustive investigation [8]; and (iii) faulty results subsisting in the published literature long after their retraction [41].

### 4.6. Limitations

In our sample, 120 respondents out of 246 (55%) indicated that the retraction of their paper was primarily not due to RDM errors, although we expected it to be based on the retraction notice. By further investigating these cases, we found that there are four main reasons why these papers are part of our original sample. First, when deciding who to contact based on the retraction notices, we aimed to include all the papers that could have been retracted due to RDM errors, even by risking the chance that we might contact authors whose paper was retracted for a different reason. Second, RDM errors are not a highly talked about concept in the published scientific literature (in contrast to research misconduct) so it is possible that the respondents did not have a clear definition of what an RDM error is. Third, previous literature showed that the retraction notices that the Retraction Watch categories are based on are often vague or misleading on purpose for the publishers to avoid later litigation. Fourth, the authors of the retracted study might not agree with the retraction or the reasons behind the retraction, even in cases of errors.

### 4.7. Openness statement

The sampling method, the survey materials, the main research questions and the data management and analysis scripts were preregistered on OSF (https://osf.io/h2ksw) before any data collection. In the following, we outline any deviations from the preregistration.

We excluded respondents who claimed their retraction was due to a data management error at the beginning of the survey but later reported that fraud happened in free text.

Also, we decided to narrow the qualitative investigation of the free-text responses regarding the authors’ experience with the retraction process or the error to two questions as the collected responses were too divergent in their content to allow for a reasonable categorization across one dimension.

Finally, we have not preregistered the figure that outlines the relationship between the most frequent causes and types of errors.

All study materials can be found at https://osf.io/h2ksw. Our analysis code and anonymized data are available at https://github.com/marton-balazs-kovacs/retractionwatch_analysis.

## Data Availability

All analysis code and anonymized datafiles are openly shared and available at GitHub [42]. Supplementary material is available online [43].
